# Therapeutic Vaccines for Tuberculosis: An Overview

**DOI:** 10.3389/fimmu.2022.878471

**Published:** 2022-06-24

**Authors:** Rania Bouzeyen, Babak Javid

**Affiliations:** Division of Experimental Medicine, University of California, San Francisco, San Francisco, CA, United States

**Keywords:** tuberculosis, mycobacterium, therapeutic vaccines, prevention of recurrence, monoclonal antibody, mRNA vaccine

## Abstract

Tuberculosis (TB), caused by *Mycobacterium tuberculosis* is the world’s deadliest bacterial infection, resulting in more than 1.4 million deaths annually. The emergence of drug-resistance to first-line antibiotic therapy poses a threat to successful treatment, and novel therapeutic options are required, particularly for drug-resistant tuberculosis. One modality emerging for TB treatment is therapeutic vaccination. As opposed to preventative vaccination – the aim of which is to prevent getting infected by *M. tuberculosis* or developing active tuberculosis, the purpose of therapeutic vaccination is as adjunctive treatment of TB or to prevent relapse following cure. Several candidate therapeutic vaccines, using killed whole-cell or live attenuated mycobacteria, mycobacterial fragments and viral vectored vaccines are in current clinical trials. Other modes of passive immunization, including monoclonal antibodies directed against *M. tuberculosis* antigens are in various pre-clinical stages of development. Here, we will discuss these various therapeutics and their proposed mechanisms of action. Although the full clinical utility of therapeutic vaccination for the treatment of tuberculosis is yet to be established, they hold potential as useful adjunct therapies.

## Introduction

Until the Covid-19 pandemic, Tuberculosis (TB), caused by *Mycobacterium tuberculosis* (Mtb) was the leading cause of death from a single infectious agent, ranking above HIV or malaria. With the disruption to public health services as a result of the Covid-19 pandemic, the number of deaths due to TB rose for the first time in over 20 years (1, 2), setting back decades of albeit slow, progress. Further threats to eradication of TB as a major public health tragedy include the rise of drug-resistant tuberculosis (DR-TB). There are over half a million cases of rifampicin-resistant TB annually. Lack of access to, and the general lower efficacy of second-line therapeutic regimens results in a disproportionate contribute of DR-TB to poor outcomes (3), and as access to newer agents such bedaquiline and delaminid increases, it will inevitably be associated with a rise of resistance to these agents also (4). It is thought that, globally, there are 19 million individuals living with latent rifampicin-resistant tuberculosis further threatening future control strategies (5, 6). In addition to the specific concerns with respect to DR-TB, and despite the current lengthy standard regimen for drug-sensitive TB (~ 6 months of multiple drugs), approximately 2-10% of patients fail initial therapy or relapse (7), necessitating re-treatment. Treatment of HIV-TB co-infection also has specific considerations, with frequent drug-drug interactions, especially with the keystone rifamycin class and worse outcomes overall. All these considerations have renewed interest in non-antibiotic treatment modalities for tuberculosis.

**Figure 1:**
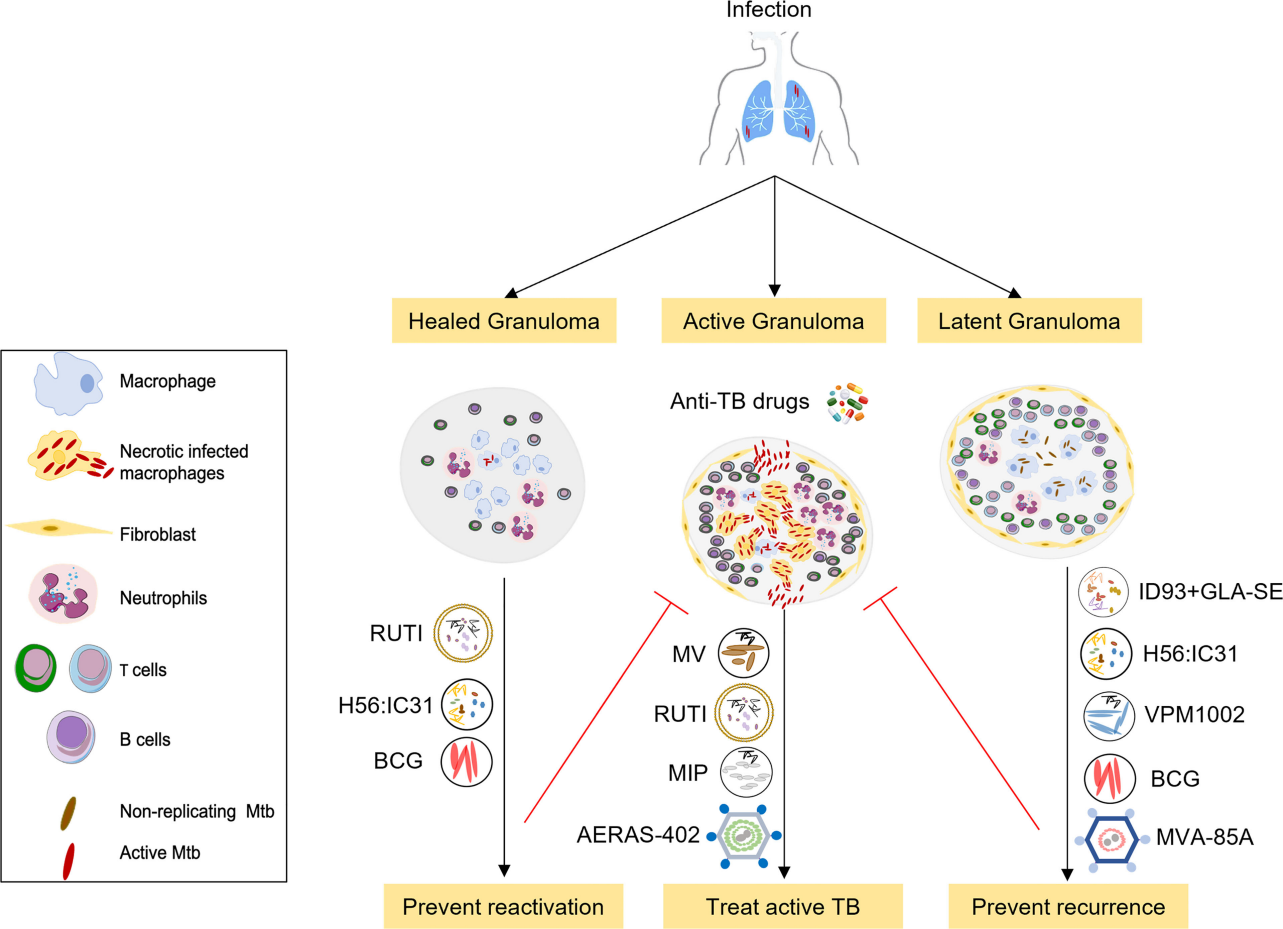
Overview of selected therapeutic TB vaccines in the clinical pipeline. The initial stages of TB infection involve inhalation of Mtb bacilli into the lung and phagocytosis by resident alveolar macrophages. The human immune system can contain or eliminate Mtb infection in the majority of cases, only a small proportion of exposed individuals go on to develop active tuberculosis. Therapeutic TB vaccines serve as immunotherapeutic adjuncts to chemotherapy and act through modulating host anti-TB immunity. These vaccines are either administrated to potentiate treatment during treatment of active disease (middle) or to prevent recurrence or relapse after standard treatment (right), or to prevent reactivation of latent tuberculosis to active tuberculosis (left). Vaccines that are being developed to improve treatment outcomes in active TB comprise *M. vaccae*, RUTI, MIP and AERAS-402. Vaccines that prevent relapse and reinfection include H56:IC31 and ID: GLA-SE subunit vaccines, RUTI, BCG, the recombinant BCG vaccine VPM1002 as well as MVA-85A. These candidates are currently in phase 2 or 3 clinical trials in TB patients during or after completion of treatment.

The usual consideration for immunization is prevention of an infection or disease. Therapeutic vaccination is when an immunomodulatory agent (the vaccine) is administered in the context of already-established disease, either to improve outcomes, shorten treatment duration, or, in the case of TB, prevent relapse (8). In 2018, the WHO defined the target product profile for therapeutic vaccines for TB. They should: (1) reduce the rate of recurrence caused by drug-sensitive and resistant TB, following completion of a full course of drug therapy; (2) increase the proportion of cured patients, in particularly for rifampicin-resistant and extensively drug-resistant TB; (3) shorten therapy duration with the goal of improving compliance and reduce the likelihood of developing drug-resistance (9) and See **Figure 1**. A further possible definition of therapeutic vaccination in the context of tuberculosis is the administration of a vaccine given to people with evidence of *M. tuberculosis* exposure, e.g. by a positive interferon-gamma release assay (IGRA+ve), to prevent progression to active tuberculosis. The WHO states that the target population for therapeutic vaccines should include all TB patients, regardless of age, drug sensitivity and co-morbidities. We will survey therapeutic vaccines currently in clinical trials (**Table 1**), as well as some potential future treatment modalities. Therapeutic TB vaccines in current clinical development fall into several categories: whole killed - and fragmented-cell vaccines; live attenuated vaccines, adjuvanted protein subunit vaccines and viral vectored vaccines.

**Table 1:** Therapeutic vaccines used in clinical trials.

Candidate and developer	Antigen, vector, and formulation	Vaccine-induced immune response	Clinical Trial statusNCT number	References
**Mycobacterial killed Whole-cell/fragmented vaccine candidates**				
***M. vaccae*** Anhui Zhifei Longcom	Whole cell, heat killed *M. vaccae*	Mixed Th1/Th2 responses Elevated Th1 response to stimulation with inactivated H37Rv High levels of IgG against Heat shock proteins	Phase III completed in patients with DS-, DR-TB and TB-HIV (NCT01977768) Phase 4 completed to compare two triple drug regimens along with immunotherapy with MV (NCT00367913)	(10) (11) (12)
**RUTI** Archivel Farma, S.L	Detoxified Mtb fragment, delivered in liposomes	Poly-antigenic Th1 response to purified mycobacterial antigens No relevant humoral responses detected	Phase II completed in LTBI patients following INH treatment ((NCT01136161) Phase II is planned to evaluate efficacy in DS- and DR-TB patients (NCT04919239, NCT05136833)	(13)
**MIP** Cadila, Indian Council of Medical Research	Whole cell, heat inactivated *Mycobacterium indicus pranii*	Elevated Th1 and humoral response in animal model Poly-antigenic response against the 16 and 38kda antigens	Phase III completed to evaluate the safety and efficacy in category I and II pulmonary TB patients (NCT002655226) (NCT00341328)	(14) (15)
**Live attenuated vaccines**				
**VPM1002** Max Plank institute Vakzine project	rBCG-expressing LLO and urease deletion	Induction of apoptosis, autophagy and inflammasome activation in macrophages Elevated frequency of CD4+ and CD8+ T cells expressing combinations of IFN-γ, TNF-α or IL-2	Phase II/III trials in adults for prevention of TB recurrence ongoing (NCT03152903)	(16) (17) (18)
**BCG**	M.bovis Bacillus Calmette-Guerin	Elevated reactive Th1 cytokine-expressing CD4, CD8, γδT cells responses and IFNγ-expressing NK cells	Phase I completed in LTBI adults for prevention of reactivation post IPT (NCT01119521)	(17, 19)
**Subunit Vaccines**				
**ID93+GLA-SE** Infectious disease research institute Quratis	RV1913, Rv2608, Rv3619, Rv3620 formulated in GLA-SE in water emulsion	Polyfunctional ID93-specific CD4+T cells Elevated levels of IgG1 and IgG3 responses to Rv1813	Phase IIa completed in adults treated TB patients for prevention of TB recurrence (NCT02465216)	(20) (21)
**H56:IC31** Statens Serum Institut, Valneva, Aeras	Ag85b, ESAT6, Rv2660c formulated in TLR9 agonist	Polyfunctional H56 specific memory CD4+T cells expressing TNF-α, and/or IL-2 and/or IFN-γ Low levels of CD8+T cell responses Increased H56-specific serum IgG levels	Phase II efficacy trial in South Africa is underway to address TB recurrence in adults who have recently treated for DS-TB (NCT03512249)	(22) (23) (24)
**Viral vectored vaccines**				
**MVA85A** **O**xford University, Aeras	Ag85A (Rv3804c, mycolyl transferase) and recombinant vaccinia virus	Persistent levels of polyfunctional CD4+ T cells expressing IL-2 and or IFN-γ	Phase 1 completed to evaluate safety and immunogenicity in latently infected individuals (NCT00456183)	(25)
**AERAS-402** Cr**ucell, AERAS**	Stable Adenoviral vector based on adenovirus 35 carries Mtb antigens Ag85A, Ag85b, and TB10.4	High levels of CD8+T bi- and monofunctional cells expressing IFNγ and or TNF-α Elevated Ad35 specific neutralizing serum	Phase II study completed to assess safety and immunogenicity in adults with active disease who are treated or on TB treatment (NCT02414828)	(26)

## Killed Whole-Cell/Fragmented Vaccine Candidates

### Mycobacterium vaccae

*Mycobacterium vaccae* (MV), originally discovered in milk and dung from cattle (hence *vaccae*, cow), is a rapidly growing environmental mycobacterium with poor pathogenic potential in humans. The vaccine was developed using a strain of *M. vaccae* isolated in a region of Uganda, where it was associated with enhanced protective efficacy of BCG immune responses to tuberculosis (27). Heat-killed preparations of *M. vaccae* (MV or SRL72) have been extensively studied as adjuncts to standard anti-tuberculous drug therapy for more than decade. It was hypothesized that inactivated MV works by stimulating the host immune responses to antigens shared with Mtb. Preclinical studies showed that mice injected with inactivated MV provoked either a predominantly Th1, or a mixed Th1/Th2 immune response, as well as potentially protective cytotoxic CD8+ T cells that could kill Mtb-infected macrophages (10, 12, 28). Administration of multiple doses series (2-5 doses) of MV, was shown to be safe and immunogenic in a Phase I and II clinical trials in HIV-infected children and adults primed or not with BCG vaccine conducted across multiple sites (29). Moreover, these studies showed that MV vaccination induced relevant immune responses in HIV-infected patients and that may be explained by the decrease of HIV loads in BCG primed and MV recipient individuals suggesting immunization with whole cell vaccine is a promising strategy for the prevention of HIV-associated TB. In an early small, randomized trial, 120 HIV seronegative newly diagnosed pulmonary TB patients were randomized to a single dose of MV versus placebo in addition to standard TB chemotherapy. MV therapy was associated with more rapid sputum culture conversion (i.e. clearance of viable Mtb), and improved radiological markers of disease (27). In addition to markers of cell-mediated immunostimulation, individuals treated with MV had increased antibody titers to the heat shock proteins HSP60 and HSP70 – which are known to be immunostimulatory (30) – in the first month of treatment, as well as raised levels of the cytokines IL-4, IL-10 and TNF-α from *ex vivo* stimulated PBMCs with inactivated Mtb (11). Of the therapeutic vaccines under consideration, MV is the most advanced in the clinical pipeline. After the initial set of small-scale studies, a single dose of intradermally delivered MV was felt to be insufficient for a robust clinical response, and trial protocols were modified to include multiple doses.

To facilitate delivery, a tablet form of heat-killed MV (termed V7) was formulated and evaluated in a small (but designated phase III) placebo-controlled efficacy trial. Patients with either drug-sensitive or drug-resistant TB were randomized to V7 or placebo daily for the first month of therapy. MV therapy was again associated with rapid sputum culture clearance of Mtb. Notably, MV therapy was also associated with weight gain in patients, and improved markers for drug-induced hepatoxicity. Given that both TB-related weight loss (31) and hepatoxicity (32) are thought to have inflammatory components, these findings are further support of the immunomodulatory effect of MV. The largest study of MV: a phase III trial conducted in China with 10,000 tuberculin skin test positive enrollees to measure efficacy in preventing progression to active tuberculosis completed enrollment in 2017 (NCT01979900) but is yet to report its findings.

In summary, MV has proven immunomodulatory activity in both animal preclinical studies as well as multiple, albeit small, clinical studies. The observed effect on decreasing time to sputum culture clearance appears robust, in multiple studies in many different patient populations. However, definitive evidence for MV in treatment outcome in large-scale studies is still awaited.

### Mycobacterium indicus pranii

MIP comprises killed *M. indicus pranii*, previously known as Mw – a non-pathogenic mycobacterium closely related to *M. avium (* 33 *)*. MIP has been studied as an adjunct therapy for leprosy and has been shown to have both immunoprophylactic and immunotherapeutic effects and the duration of multi-drug therapy in leprosy patients by improving the immune responses to *M. leprae* (34). In keeping with other whole-cell mycobacterial vaccines, preclinical evaluation was confirmed to reduce organ Mtb bacillary burden in small animal models, associated with increased early cell-mediated immunity, including, notably, increased cytotoxic T cells and followed by a balanced Th1/Th2 responses in later part of the chemotherapy (14).

Clinical studies have focused on “category II TB”, i.e. patients that have either failed therapy, relapsed, or otherwise been lost to follow-up. These patients have high rates of treatment failure, as well as risk to progression to drug-resistant TB (15). In a phase II study of 890 sputum smear positive category II patients, patients were randomized to 6 injections of MIP or placebo. Unfortunately, 248 patients (equally distributed between the intervention and control arms) failed to complete the treatment regimen and were excluded from per protocol analysis. There was a subtle decrease in time to sputum culture conversion in the MIP arm. However, there was no statistically significant different in cure rates – possibly because the high rates of cure (94% in MIP arm and 90% in placebo arm) meant the study was underpowered to detect a small difference. In the two years of follow-up, a total of 34 patients in the MIP arm and 26 in the placebo arm relapsed, which again, was not statistically significant (15). There is an ongoing phase III efficacy and safety trial for preventing pulmonary TB among healthy-household contacts of sputum positive TB patients in India and first results are expected soon (18). In summary, MIP has shown robust evidence of immunostimulation. Evidence of efficacy, however, is still lacking or awaited in either treatment, or prevention of active TB following high risk exposure.

### RUTI

RUTI is composed of liposomes containing detoxified fragments of Mtb that was cultured under conditions of hypoxia/stress, which are known to induce expression of a wide-range of stress-related proteins (35). The rationale is that by inducing an immune response to these antigens, RUTI will have improved efficacy against non-replicating bacteria (NRB), that are hypothesized to contribute to the requirement of prolonged treatment, as well as relapse (36).

In endemic settings, populations at increased risk for latent TB infection can be treated using Isoniazid Preventive Therapy (IPT) for 6-12 months. Although IPT has been demonstrated to reduce the risk of reactivation by 70% in high-risk groups, later studies have shown waning protection as soon as 18 months after short-term INH based regimens in high endemic settings (37). This could be due to the low adherence rates, or re-infection. It is thought that Mtb bacilli in individuals with latent infection survive within foamy macrophages in necrotic granulomata that represent an important immunosuppressive barrier (38). Antibiotic treatment is required to kill replicating bacteria as well as eliminate the outermost layer of foamy macrophages and may allow the egress of new phagocytes. Administration of RUTI in this context was proposed to induce a broad of T cells response that would prevent the reactivation of, or kill the remaining NRB (35).

In small animal studies, administration of RUTI to Mtb-infected guinea pigs and mice, following incomplete chemotherapy resulted in increased Mtb-specific CD4+and CD8+ cellular responses and antibody production, and was associated with decreased bacillary organ burden and lung pathology (39). Furthermore, reduction in post-chemotherapy relapse was reported, in which passive immunization with sera, obtained from mice treated with RUTI, exerted significant protection against reactivation of Mtb infection in SCID mice (40). In human studies, a combined phase I/II clinical trial was conducted to test the tolerability and immunogenicity of different RUTI doses in HIV positive and negative patients after completion of one-month INH treatment (13, 41). The results demonstrated that RUTI vaccination was tolerated and that one inoculation of 25μg of RUTI resulted in poly-antigenic responses, especially against the 16 kDa and 38 kDa antigens, which are biomarkers associated with LTBI. There are no reported efficacy data for RUTI, but small phase II trials (NCT04919239, NCT02711735, NCT05136833) are currently underway to evaluate RUTI as adjunctive therapy for drug sensitive and rifampicin-resistant tuberculosis.

## Live Attenuated Vaccines

### VPM1002 (BCG ΔureC::hly)

VPM1002 is recombinant BCG-ΔureC::hly, i.e. in which the urease C gene, *ureC* has been deleted and replaced with the listeriolysin O (LLO) gene from *Listeria monocytogenes*. Urease was known to reduce phagolysosome acidification and promotes mycobacterial survival (17) Listeriolysin O is a thiol-activated, cholesterol-dependent cytolysin that is only active in acidic environments. Hence deletion of *ureC* would increase LLO activity (17). In *Listeria*, LLO is absolutely required for virulence (42), and functions to disrupt the phagosome membrane to allow pathogen escape to the cytosol (43). It has now been demonstrated that Mtb can disrupt the phagosome membrane, in an Esx-1-dependent mechanism (44). Esx-1 is a type VII secretion machinery that is essential for Mtb virulence (45, 46) and is missing in BCG. Hence BCG, unlike Mtb, cannot engage in phagolysosomal escape. By expressing LLO, BCG acquires phagolysosomal escape *via* a different mechanism, and consequently improves antigen accessibility, in particular for loading onto MHC class I. VPM1002 was demonstrated to enhance both innate and adaptive immunity in animal models, in part *via* inducing apoptosis, autophagy and stimulation of the NRLP3 inflammasome. Compared with wild-type BCG, VPM1002 also resulted in greater stimulation of humoral immunity and germinal center activation (17). Although the initial clinical studies for VPM1002 have been as a candidate preventative vaccine, one animal study suggests potential as a therapeutic vaccine. Mice were infected with Mtb and then treated with antibiotics for 40 days until viable Mtb was no longer recovered from organs. Mice were then treated with BCG, VPM1002 or saline. When organ bacterial burdens were evaluated at 250 days post initial infection, VPM1002 was clearly superior to BCG or saline, although no mice achieved sterile cure (16). A phase II/III clinical trial with VPM1002 is currently ongoing in India to for the prevention of recurrence of active TB in adults following completion of standard therapy (NCT03152903).

### Bacillus Calmette Guérin

Bacillus Calmette-Guerin, a live attenuated strain of *Mycobacterium bovis* (*M. bovis* is part of the *M. tuberculosis* complex and responsible for cattle TB), is the only licensed TB vaccine and was developed a century ago. It is the world’s most extensively used vaccine. BCG has a wide variation in reported efficacy as a preventative vaccine. Despite expert opinion that BCG may be ineffective as a therapeutic in established active TB infection, possibly because active infection masks BCG-induced immunity (47), several animal studies have suggested that BCG may enhance antibiotic activity in experimental *M. tuberculosis* infection. In an early study, Dhillon and Mitchinson showed that BCG immunization paradoxically decreased the bactericidal activity of isoniazid in Mtb-infected mouse spleens, but not lungs, and had no effect on rifampicin-mediated killing. However, in the guinea-pig model, BCG increased the activity of both drugs in both lungs and spleens (48). In another guinea-pig study, animals immunized +/- BCG were infected with *M. tuberculosis* and then treated with a combination of rifampicin, isoniazid and pyrazinamide (RHZ), the core of the standard TB regimen. Although BCG immunization had no additional effect on bacterial organ burden on top of antibiotics, it did prolong survival of the infected animals compared with drug-treatment alone and the combination of BCG and drug treatment was also superior to antibiotics alone in reducing pathology (49).

In the context of clinical studies, a secondary analysis of either BCG or rifampicin alone or in combination for the prevention of leprosy transmission in 21,711 close contacts of 1037 index leprosy patients was performed. The original study was designed to determine the efficacy of rifampicin (against placebo), but the observation that ~40% of participants had a history of BCG vaccination given at infancy (well-balanced in both arms of the trial, although in both arms, a larger proportion of young enrollees had a history of BCG vaccination) allowed for the secondary analysis. BCG given at infancy and rifampicin post contact exposure (in the absence of BCG) had an efficacy of 57 and 58% respectively for prevention of leprosy in contacts. However, the combination of BCG and rifampicin had an increased efficacy of 80% (50). Although no efficacy data are available for TB, BCG revaccination is safe and well tolerated in individuals with evidence of LTBI (51). In a phase I study of 82 HIV-ve adults with a strongly positive tuberculin skin test, indicative of LTBI, and who had visible evidence (by scar) of prior BCG vaccination, participants were randomized either to isoniazid preventative therapy (IPT) followed by BCG revaccination, or BCG revaccination followed by IPT (19). IPT had negligible impact on mycobacteria-specific T cell responses. However, BCG revaccination (regardless of IPT order) was associated with the stimulation of a durable, BCG-reactive NK cell response, which was also detected in BCG-immunized infants (19). Given that BCG revaccination was associated with a moderate reduction in primary infection with *M. tuberculosis* (52), the relevance of these BCG-reactive NK cells on restricting *M. tuberculosis*, and whether they are relevant in the context of active or recently treated TB warrant further investigation.

## Subunit Vaccines

### ID93+GLA-SE

ID93 is comprised of four Mtb proteins expressed in tandem. The candidate antigens were identified in a screen of immunodominant human T cell epitopes that were also immunogenic in mice (53). ID93 is formulated with a synthetic toll like receptor 4 (TLR-4) agonist in a stable oil-in water emulsion known as glucopyranosyl lipid A stable emulsion (GLA-SE). In initial studies as a preventative vaccine either alone or as a booster to priming by BCG, ID93-GLA-SE vaccination induced robust polyfunctional Th1 responses, characterized by antigen-specific IFNγ, TNF-α and IL-2 CD4+ T cells, and was superior to BCG alone in both mice and guinea-pigs (54). In a subsequent study, ID-93+GLA-SE was evaluated for therapeutic vaccination in a mouse model as well as cynomologous macaques. The murine studies used SWR/J mice that, unlike the more widely used C57/BL6 mice do not develop chronic infection, but instead progressively sicken and die after ~100 days unless rescued with antibiotic therapy. Adjunctive treatment with vaccination both increased survival of the mice and shortened the length of antibiotics required to rescue the mice (20). Treatment of the non-human primates with 1 month of rifampicin and isoniazid was insufficient to completely resolve infection when given alone, but when combined with three doses of ID-93+GLA-SE following antibiotic treatment, resolved radiographic evidence of disease (20). Building on these studies, ID-93+GLA-SE has been evaluated in an early-stage phase IIa study for prevention of recurrence/relapse in HIV-positive patients who had recently completed a four-month course of standard chemotherapy for pulmonary tuberculosis (21). The vaccine was well tolerated and induced antigen specific CD4-T cell responses in stimulated whole blood and PBMCs. Moreover, IgG-ID93 specific responses were predominantly IgG1 and IgG3. Since only 61 patients were evaluated in all arms, the study was not powered for efficacy (21).

### H56:IC31

H56:IC31 is a recombinant fusion protein comprised of three Mtb antigens. The vaccine is formulated in a stabilizing agent containing a TLR-9 agonist as adjuvant and designed to drive Th1 immune response against replicating and non-replicating bacilli (6). H56:IC31 has been shown to prevent Mtb reactivation in nonhuman primates (55, 56). A phase 1 clinical trial, carried out with 25 adults, HIV negative, with or without latent TB infection, showed that H56 vaccine induced a polyfunctional CD4+T cells with memory phenotype that persisted for up to 210 days post vaccination (22). Following evidence of both safety and immunogenicity in a phase I study conducted in HIV negative adults who had recently been successfully treated for drug-sensitive pulmonary TB (23), H56:IC31 underwent a phase I/II study to evaluate it as a host-directed therapy alone or in combination with the cyclooxygenase-2 inhibitor (COX2i) etoricoxib. COX inhibition had been shown to both reduce or exacerbate experimental Mtb infection (57). H56:IC31 was safe and immunogenic, as was etoricoxib, but the combination of vaccination with COX2i resulted in blunted immune responses, suggested the two may be antagonistic as host-directed therapies (58). As with ID93, efficacy data is not yet available, although a phase II study of 900 HIV negative TB patients who have recently completed treatment is currently underway (NCT03512249).

### M72/AS01_E_

M72 is a subunit vaccine comprising a recombinant fusion protein of two Mtb-derived proteins, Mtb32A and Mtb39A; delivered with the adjuvant AS01_E_, which has been used in the clinically approved malaria vaccine RTS,S as well as the zoster vaccine Shingrix. A phase IIb trial of 3573 adult participants that were HIV-ve and IGRA+, of whom 3289 were included in the per-protocol efficacy calculation, received either the vaccine or placebo. A total of 39 cases of active tuberculosis were detected in the participants, of which 13 were in the vaccine arm, resulting in a vaccine efficacy of ~50% (59). This was the first demonstration of vaccine efficacy for tuberculosis against development of active TB in the post BCG era, and validated that protein subunit vaccines, which have been developed successfully against viral and extra-cellular bacterial (e.g. the acellular pertussis vaccine) infections, could potentially be effective against an intracellular pathogen. Although the mechanisms of protection are not known, both CD4+ T cell and antibody responses were associated with protection (59).

## Viral-Vectored Therapeutic Vaccines

One potential silver lining from the Covid-19 pandemic has been the remarkable progress in novel vaccine platforms. For the first time, both viral- and mRNA-delivered subunit vaccines (against SARS-CoV-2 spike protein) have not only been evaluated in clinical trials but found to be effective and approved (60). Although mRNA vaccine platforms are only now starting to be investigated for TB, virus-delivered subunit vaccines have been investigated in both preclinical and clinical studies.

### Modified Vaccinia Ankara 85A

MVA is a viral vector comprising a recombinant replication-deficient modified vaccinia virus, Ankara (MVA) that allows insertion of large immunogenic sequences, up to 10kbp, and is efficient at induction of specific T cell responses. Given their safety and immunogenicity profile, MVA vectors have been extensively tested as prophylactic vaccine platform in endemic settings (61). MVA expressing antigen 85A (MVA85A) was shown to induce a robust Ag85A-specific CD4+ and CD8+ T cell responses and protected animals to the same extent as BCG following low-dose aerosol infection (62). The first phase 1 study using MVA85A was performed in healthy volunteers and was found to promote polyfunctional CD4+ T cells, likewise, the vaccine was deemed to be safe in infants, HIV positive individuals and LTBI subjects in endemic populations from Gambia, South Africa and Nigeria (63–65). MVA85A completed phase II clinical trials to evaluate its safety and protective effect in a prime boost regimen in infants of HIV infected mothers followed by selective BCG boost at 8 weeks for HIV negative infants (NCT01650389). The data showed that MVA85A prime vaccination of HIV uninfected newborns was safe and induced a modest antigen specific immune response. A phase I clinical study in UK to investigate the effect of MVA85A, administrated by the aerosol inhaled or intramuscular route in healthy LTBI+ was terminated due to challenges with recruitment in the UK sites (NCT02532036). However, the vaccine has completed a small non-randomized phase I study to evaluate its safety and immunogenicity in 12 healthy volunteers latently infected with TB (NCT00456183) (25). Although this trial was limited by the heterogeneity of the population used and the small sample size, it showed that MVA85A is immunogenic in LTBI individuals as well as in BCG vaccinated but Mtb-uninfected individuals. Moreover, the vaccine induced a persistent Ag85A-specific polyfunctional T cell response until week 24 post vaccination. MVA85A is yet to be tested as an adjunct to standard drugs in MDR/XDR subjects, however in a chronic post-exposure mouse model, it was shown that combination of an antibiotic regimen with MVA harboring ten Mtb antigens (MVATG18598), each representative of different phases of TB disease, induced a robust Th1 response and improved the efficacy of chemotherapy (66).

### Adenovirus

Adenoviruses remain the most widely utilized platform for vaccine design, given their safety, high efficiency gene transduction and induction of robust immune responses. Human adenovirus serotype 35 (AdHu35) has been used extensively as a preventative vaccine (AERAS-402). The vaccine is a replication deficient adenovirus serotype 35 containing DNA encoding fusion proteins of three Mtb antigens: Ag85A, Ag85B and TB10.4. Both mucosal (intranasal) and intra-muscular administration of AERAS-402 conferred protective immunity against Mtb infection in mice (67). However, despite eliciting a robust immune response in immunized macaques, aerosol administration of AERAS-402 failed to protect the primates from high-dose (~275 colony forming units) challenge with *M. tuberculosis* (68). To determine the safety and immunogenicity of AERAS-402 in TB patients and to exclude the possibility of vaccine-induced immunopathology, a dose-escalation study in 72 adults who were either in the middle of treatment of active tuberculosis or had recently completed treatment was performed. Overall, the vaccine was well-tolerated and highly immunogenic. One study participant died, but from unrelated causes (broncheoalveolar carcinoma) (26). Nonetheless, immunogenicity does not imply functional immune responses. In a study of BCG-primed, AERAS-402 boosted participants, robust antigen-specific CD4+ and CD8+ T cells were identified in the vaccinated arm. However, the cytotoxic (CD8+) T cells were unable to recognized Mtb-infected dendritic cells (69). These findings are in keeping with other studies suggesting that *M. tuberculosis* engages in evasion of immunity, possibly *via* immunodominant responses (70), which further complicates effective vaccine design and discovery. To our knowledge, AERAS-402 is no longer in active clinical development.

Studies with a different adenoviral vector, this time chimpanzee adenovirus serotype-68 (ChAd-68) expressing Mtb antigen 85A was effective as adjunct to chemotherapy in the Balb/C mouse model of infection – but only when given intra-nasally and not when given intra-muscularly (71). These data suggest that route as well as formulation of viral-vectored vaccines may be an important consideration of potential efficacy.

### Passive Therapeutic Immunization With Monoclonal Antibodies?

Vaccines: whether cell- or subunit-based, deliver immunogens that then evoke potentially protective immune responses. Alternatively, passive transfer of immune effectors, such as antibodies, can bypass the need to elicit an appropriate immune response. Recombinantly expressed monoclonal antibodies (mAbs) have been used extensively for non-infectious diseases, but their use in infection prior to Covid-19 had been restricted to a few instances such as for respiratory syncytial virus (72), in part due to the cost of goods and manufacture. The efficacy of virus-specific and neutralizing mAbs for Covid-19, especially if given early in onset of the disease has now been amply demonstrated. However, translating these Covid successes to TB is not entirely straightforward. For decades, there was controversy with regards to the potential role of antibody-mediated immunity to TB. Recent evidence, from studies of human antibodies specific for Mtb, suggest that antibodies may well be protective in TB (reviewed in (73)). However, unlike viral infections, where neutralization of virus is the single most, albeit not complete (74) mechanism mediating antibody efficacy, protective anti-TB antibodies rely on Fc-mediated effector functions (75, 76). Since recombinant mAbs may have different Fc properties than naturally occurring antibodies, for example with regards to glycosylation (77), mAbs may possess different effector functions than the original natural antibodies from which they are derived. We recently demonstrated that recombinant mAbs directed against a subunit of the Mtb phosphate transporter were still protective both in human *ex vivo* as well as murine assays (76), suggesting that recombinant mAbs may be potentially useful tools. Nonetheless, treatment of an acute respiratory infection (i.e. Covid-19) and a chronic pulmonary disease such as TB are not directly comparable, not least due to the requirement for repeated dosing and the associated increased costs. There is also the potential for detrimental immunopathology that would need to be investigated carefully in both preclinical, and eventually, clinical studies.

## Concluding Remarks

The idea of therapeutic immunization is not new. Convalescent plasma/serum therapy was one of the most widely used therapeutics for infectious diseases prior to the discovery of antimicrobial drugs (78, 79). There are a plethora of data to support therapeutic vaccination from preclinical models of tuberculosis. Moreover, trials to assess efficacy of therapeutic vaccines are easier and less costly to design than for preventative vaccines, since they can use much smaller numbers of enrollees due to higher event rates. Despite this, hard evidence supporting a role for therapeutic vaccination for clinically-relevant endpoints such as cure and relapse are still lacking. What is clear is that therapeutic vaccination can influence host immune responses. Given that even in cured TB patients, long-term sequelae of disease due to immunopathology is present in a substantial proportion of patients (80, 81) revisiting therapeutic vaccination through the lens of modifying post-cure pathology may offer a new path for using these reagents for treatment of the world’s deadliest bacterial disease.

## Author Contributions

RB and BJ conceived and wrote the manuscript together. All authors contributed to the article and approved the submitted version.

## Funding

This work was supported in part by INV-038660 from the Bill & Melinda Gates Foundation. BJ is an Investigator of the Wellcome Trust (207487/C/17/Z).

## Conflict of Interest

The authors declare that the research was conducted in the absence of any commercial or financial relationships that could be construed as a potential conflict of interest.

## Publisher’s Note

All claims expressed in this article are solely those of the authors and do not necessarily represent those of their affiliated organizations, or those of the publisher, the editors and the reviewers. Any product that may be evaluated in this article, or claim that may be made by its manufacturer, is not guaranteed or endorsed by the publisher.
